# Natural antisense transcripts in the biological hallmarks of cancer: powerful regulators hidden in the dark

**DOI:** 10.1186/s13046-020-01700-0

**Published:** 2020-09-14

**Authors:** Shanshan Zhao, Xue Zhang, Shuo Chen, Song Zhang

**Affiliations:** 1 Key Laboratory of Reproductive Dysfunction Diseases and Fertility Remodeling of Liaoning Province, Reproductive Medicine Center, Obstetrics and Gynecology Department, Shengjing Hospital Affiliated to China Medical University, 110022 Shenyang, Liaoning China; 2 Department of Epigenetics, China Medical University, 110122 Shenyang, Liaoning China; 3 Department of Gynecologic Oncology Research Office, The Third Affiliated Hospital of Guangzhou Medical University, 510150 Guangzhou, Guangdong China; 4 Department of Thoracic Surgery, The First Affiliated Hospital of China Medical University, 110001 Shenyang, Liaoning China; 5 Department of Environmental and Occupational Health, School of Public Health, China Medical University, 110122 Shenyang, Liaoning China

**Keywords:** Natural antisense transcripts, NATs, Cancer, Hallmarks of cancer

## Abstract

Natural antisense transcripts (NATs), which are transcribed from opposite strands of DNA with partial or complete overlap, affect multiple stages of gene expression, from epigenetic to post-translational modifications. NATs are dysregulated in various types of cancer, and an increasing number of studies focusing on NATs as pivotal regulators of the hallmarks of cancer and as promising candidates for cancer therapy are just beginning to unravel the mystery. Here, we summarize the existing knowledge on NATs to highlight their underlying mechanisms of functions in cancer biology, discuss their potential roles in therapeutic application, and explore future research directions.

## Background

High-throughput RNA sequencing has revealed the universal transcriptional information of the human genome, of which non-coding sequences account for 98% [1]. Non-coding sequences were previously considered as “junk DNA” due to their low expression levels, unknown functions, and heterogeneity [2, 3]. However, mounting evidence has demonstrated that non-coding RNAs (ncRNAs) can modulate the expression of protein-coding RNA and play indispensable roles in various biological processes, including tumorigenesis, metastasis, and therapeutic resistance [4–7].

ncRNAs are classified into two groups based on the transcript length. In general, 200 nucleotides is used as the biophysical threshold for separating long ncRNAs (lncRNAs) from short ncRNAs. The lncRNAs are further divided into different subclasses, and include intronic lncRNAs, bidirectional lncRNAs, intergenic lncRNAs, enhancer RNAs, circular RNAs (circRNAs), pseudogenes, sense lncRNAs, and natural antisense transcripts (NATs), based on their original genomic locations or their relationships with protein-coding genes. For more details on lncRNAs classification, please refer to [8, 9].

NATs are RNA molecules transcribed from the opposite strands of DNA that partially or completely overlap with the sense RNA [10, 11]. Investigations of NATs have been initiated in recent years due to the development of high-throughput sequencing, and NATs have attracted increasing interest due to their significant roles in diverse pathophysiological processes [11]. Many NATs were first annotated as lncRNAs, and therefore described as a subclass of lncRNA [12, 13]. Even so, NATs can also be short ncRNAs [11, 14] or protein-coding genes [15]. The incomplete overlap between the classification of NATs and lncRNAs may be confusing. Therefore, it is necessary to understand the generation and intrinsic characteristics of NATs before investigating their potential mechanisms and functions.

NATs can be produced from shared bidirectional promoters (i.e., when the transcription start sites (TSS) of a NAT and its sense counterpart are separated by ≤ 1 kb [16]), independent, or latent promoters found within genes (i.e., when the NAT TSS is entirely covered by its sense counterpart [17]). The expression of NAT is also regulated by promoters and enhancers [12, 17]. Furthermore, NATs can be capped, poly-adenylated and undergo intron excision, much like the maturation process of mRNAs [18]. Based on the orientation with reference to sense genes, NATs can be divided into three categories: (1) head-to-head (5’ -regions overlap), (2) tail-to-tail (3’ -regions overlap), and (3) embedded (one transcript is completely incorporated within the other) [17].

**Fig. 1:**
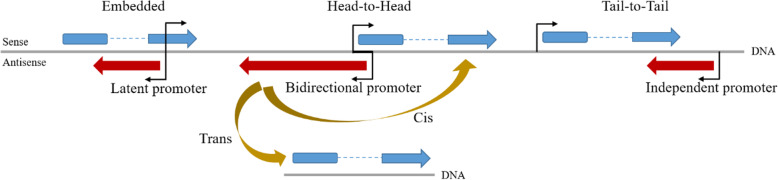
NAT structural features and action models. NATs may originate from shared bidirectional promoters, independent, or latent promoters found within genes. Based on the orientation with reference to sense genes, NATs can be classified as head-to-head (5′-regions overlap), tail-to-tail (3′-regions overlap), or embedded (one transcript is completely incorporated within the other). Based on the action model, NATs can also be divided into cis-NATs and trans-NATs

NATs can also be divided into cis-NATs, transcribed from the same genomic loci on different strands with perfect RNA-RNA sequence complementarity, and trans-NATs, transcribed from different genomic loci with imperfect sequence complementarity (Fig. 1) [10, 19].

Functionally, NATs participate in multiple processes of gene expression regulation, from controlling epigenetic modifications to modulating post-transcriptional modifications [11, 20]. Aberrant expression of NATs is associated with human disease pathology, including cancer [21]. The purpose of this review is to classify the molecular mechanisms of NATs in cancer and their roles in tumorigenesis and progression.

### Introduction to NATs in cancer

The term cancer refers to a group of genetic diseases, in which cell signaling networks are modified, resulting in disorganized cell homeostasis and uncontrolled growth [22, 23]. As non-coding DNA accounts for 98% of the genome, it seems inevitable that aberration in the non-coding genome serves a role in regulating the phenotypes of cancer [2]. Accumulating evidence has demonstrated that NATs act as biomarkers for cellular pathophysiologies, provide diagnostic and prognostic value, and reveal promising therapeutic targets for cancer [11, 12]. For example, as a classic oncogenic NAT, HOTAIR is situated in the HOXC locus and transcribed from the antisense strand [24, 25]. HOTAIR supports tumor-cell stemness, proliferation and metastasis, as well as therapeutic resistance in 26 human tumor types, by interacting with molecules such as miRNAs, ubiquitin ligases, and chromatin modifiers [26–32]. Additionally, HOXA11-AS, the homeobox A11 antisense transcript, acts as an initiator of tumorigenesis and metastasis through epigenetic modifications in the nucleus or as an miRNA sponge (RNA molecules that act as competing endogenous RNAs (ceRNAs) by binding to miRNAs, thereby reducing the regulatory effects of miRNAs on target mRNAs, subsequently improving mRNA stability and protein expression [33]) in the cytoplasm [34].

NATs may also participate in tumorigenesis and progression via interplay with paired sense RNA in cis and/or in trans. ZEB1-AS1, which is transcribed from the antisense strand of the ZEB1 locus, correlates positively with ZEB1 expression and promotes multiple malignant phenotypes in numerous malignancies [35–42]. ZEB1-AS1 can bind and recruit histone methyltransferase MLL1 or histone acetyltransferase p300 to the ZEB1 promoter region, activating ZEB1 transcription epigenetically, thereby acting as an oncogene in cis [41, 43]. ZEB1-AS1 can also facilitate colon adenocarcinoma progression in trans by sponging miR-455-3p to upregulate PAK2, which is tightly associated with tumor cell proliferation, invasion, and apoptosis [44–46]. These are just introductions to NATs in cancer; the following sections will provide more detail.

### Molecular mechanisms of NAT activity

Different subcellular localizations of NATs bring about different mechanisms of biological functions. NATs accumulated in the nucleus are mainly involved in epigenetic modifications, transcriptional interference, alternative splicing, and RNA processing. Whereas cytoplasmic NATs mainly regulate RNA stability and/or mRNA translatability [2, 11]. Therefore, we have divided NATs into nuclear NATs and cytoplasmic NATs (Table 1). Each category will be discussed in detail below.

**Table 1:** NATs in the hallmarks of cancer

NAT	Preferential localization	Cancer type	Mechanism	Hallmark	Ref.
HOTAIR	Nucleus/cytoplasm	Various types	Epigenetic modifications miRNA sponge PTM	Resistance to cell death Activation of invasion and metastasis Genome instability and mutation Tumor-promoting inflammation	[29, 31, 125, 165]
HOXA11-AS	Nucleus/cytoplasm	Various types	Epigenetic modifications miRNA sponge	Sustaining proliferative signaling Activation of invasion and metastasis Tumor-promoting inflammation	[34, 168, 187]
ZEB1-AS1	Nucleus/cytoplasm	Various types	Epigenetic modifications miRNA sponge	Sustaining proliferative signaling Resistance to cell death Activation of invasion and metastasis	[40, 42, 46]
KHPS1	Nucleus	Various types	Epigenetic modification	Resistance to cell death Tumor-promoting inflammation Reprogramming energy metabolism	[51, 188, 189]
NAT of PTEN	Nucleus	Various types	Epigenetic modification	Evasion of growth suppressors	[56]
AIRN	Nucleus	Various types	Transcriptional interference	Evasion of growth suppressors	[59, 60, 190]
GNG12-AS	Nucleus	Breast cancer	Transcriptional interference	Resistance to cell death Activation of invasion and metastasis	[63]
EGOT	Nucleus	Breast cancer	Alternative splicing	Resistance to cell death	[67]
ZEB2-AS1	Nucleus/cytoplasm	Various types	Alternative splicing promotes mRNA stability and translatability PTM	Sustaining proliferative signaling Activation of invasion and metastasis	[68, 69, 191]
PCA3	Nucleus	Prostate cancer	RNA editing	Evasion of growth suppressors	[71]
HIF1A-AS2	Nucleus/cytoplasm	Various types	RNA editing miRNA sponge	Induction of angiogenesis Tumor-promoting inflammation Reprogramming energy metabolism	[74, 136, 137]
GLS-AS	Cytoplasm	Pancreatic cancer	RNAi	Sustaining proliferative signaling Activation of invasion and metastasis Reprogramming energy metabolism	[80]
PDCD4-AS1	Nucleus	Breast cancer	promotes mRNA stability	Activation of invasion and metastasis	[82]
KRT7-AS1	Nucleus	Gastric cancer	promotes mRNA stability	Sustaining proliferative signaling Activation of invasion and metastasis	[83]
DHPS	Nucleus/cytoplasm	Gastric cancer	promotes mRNA stability	Sustaining proliferative signaling	[15]
NAT of BCMA	Cytoplasm	Mlultiple myeloma	Translational interference	Resistance to cell death Tumor-promoting inflammation	[84, 192]
NAT of PU.1	Cytoplasm	Leukemia	Translational interference	Enabling replicative immortality	[86, 193]
ZFAS1	Nucleus/cytoplasm	Osteosarcoma,colorectal cancer	Epigenetic modification miRNA sponge	Sustaining proliferative signaling Activation of invasion and metastasis Induction of angiogenesis	[89, 139]
SOX9-AS1	Cytoplasm	Hepatocellular carcinoma	miRNA sponge	Sustaining proliferative signaling Activation of invasion and metastasis	[90]
ASB16-AS1	Cytoplasm	Gastric cancer	PTM	Sustaining proliferative signaling Resistance to cell death	[95]
RHPN1-AS1	Cytoplasm	Cervical cancer	miRNA sponge	Activation of invasion and metastasis	[104]
MYLK-AS1	Cytoplasm	Hepatocellular carcinoma	PTM	Sustaining proliferative signaling Activation of invasion and metastasis	[105]
PANDAR	Nucleus	Ovarian cancer	Alternative splicing PTM	Evasion of growth suppressors	[109]
WRAP53α	Nucleus/cytoplasm	Breast cancer	promotes mRNA stability	Evasion of growth suppressors Resistance to cell death	[110]
FOXD2-AS1	Cytoplasm	Thyroid cancer	miRNA sponge	Enabling replicative immortality	[113]
TP73-AS1	Nucleus	Clear cell renal cell carcinoma, ovarian cancer	Epigenetic modification	Resistance to cell death Activation of invasion and metastasis	[120, 129]
HAGLROS	Cytoplasm	Colorectal cancer	miRNA sponge PTM	Resistance to cell death	[121]
HOXD-AS1	Cytoplasm	Lung cancer	miRNA sponge	Sustaining proliferative signaling Activation of invasion and metastasis	[128]
RAD51-AS1	Cytoplasm	Hepatocellular carcinoma	Translational interference	Genome instability and mutation	[145]
WRAP53β	Cytoplasm	Various types	PTM	Genome instability and mutation	[167]
SIRT1-AS	Cytoplasm	Hepatocellular carcinoma	miRNA sponge	Activation of invasion and metastasis Genome instability and mutation	[151]
NKX2-1-AS1	Nucleus	Lung cancer	Binding decoy	Avoidance of immune detection and destruction	[154]
ITIH4-AS1	Cytoplasm	Colorectal cancer	PTM	Tumor-promoting inflammation	[169]
IDH1-AS1	Cytoplasm	Cervical cancer	Physical interference	Reprogramming energy metabolism	[176]

### Epigenetic modifications

NATs can control transcription initiation via histone modifications, which mainly consist of methylation and acetylation, or methylation in the CpG islands of the promoter [11, 47]. A classic example of NAT-mediated histone modification is HOTAIR. Mechanically, the 5′-region of HOTAIR recruits PRC2, an epigenetic silencer of target genes that catalyzes the trimethylation of histone H3 lysine 27 (H3K27me3), facilitating epigenetic gene silencing [48].

**Fig. 2:**
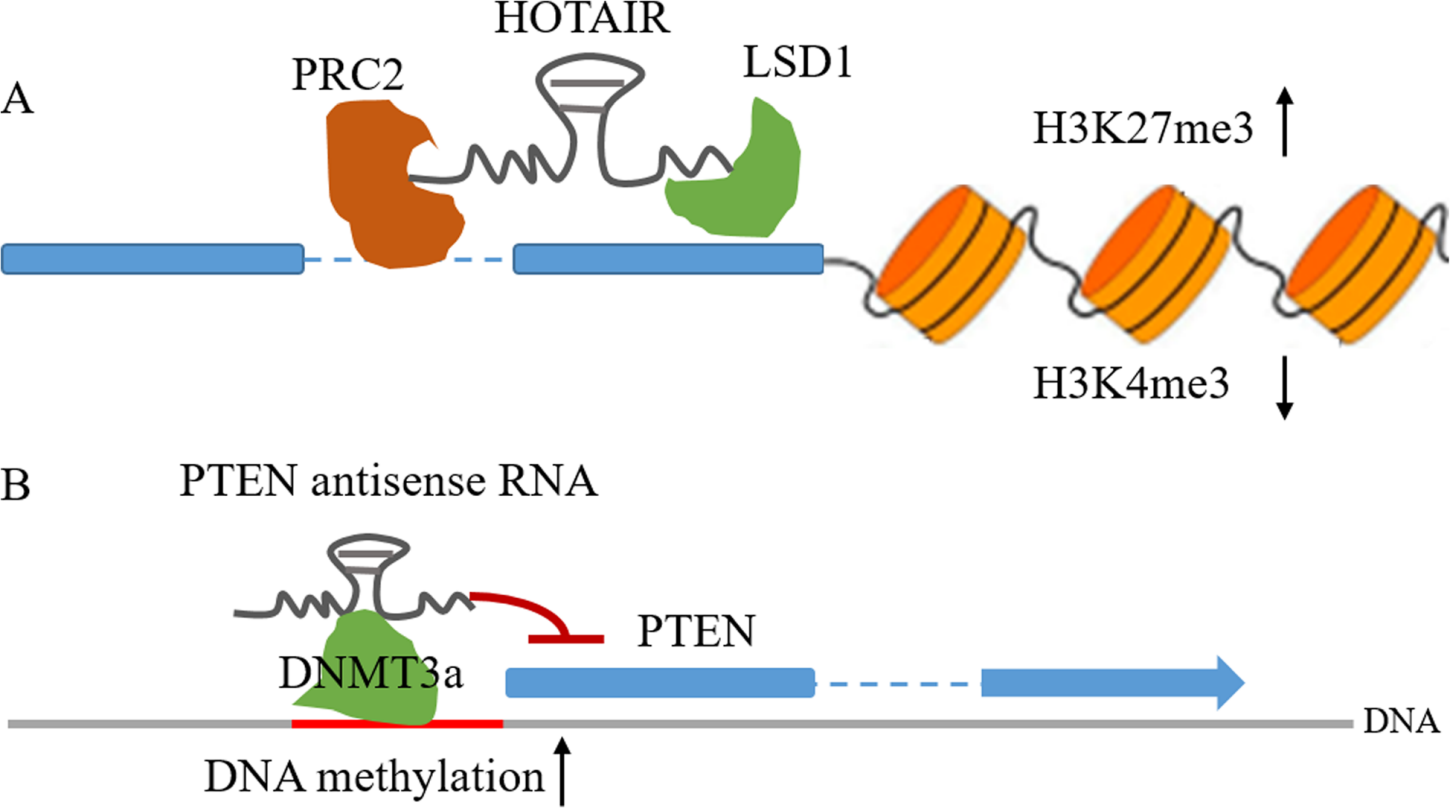
NATs in epigenetic modifications. **a**HOTAIR acts as a scaffold to tether the PRC2 and LSD1 complex and mediates H3K27 trimethylation and H3K4 demethylation. **b** NATs can interact, directly or indirectly, with DNA methyltransferases (DNMT3a) and guide DNA methylation, resulting in the repression of sense RNA transcription

Moreover, the 3′-region of HOTAIR can also recruit another chromatin regulator, LSD1, a histone demethylase that triggers histone H3 lysine K4 demethylation and acts as a histone mark for gene repression [49]. Therefore, HOTAIR can function as a bifunctional regulatory RNA or a molecular “scaffold” to alter the chromatin state, leading to transcription repression and epigenetic silencing of the target genes [48, 50] (Fig. 2a). Taken together, this evidence proposes a model in which NATs facilitate recruitment of chromatin-modifying complexes when located at the appropriate sites.

NATs also play potential roles in mediating gene activation. It has been suggested that NATs can function as a ‘‘local address code’’ and thus establish a DNA-RNA triplex structure with target chromatin-modifying enzymes at specific gene sequences [41]. For example, KHPS1, which is transcribed from the antisense strand of proto-oncogene SPHK1, can form an RNA-DNA triplex at the SPHK1 enhancer. This triplex then recruits CBP/p300 to the SPHK1 promoter. CBP and p300 are closely related histone acetyltransferases (HATs) that act together as a cis-regulatory element to increase H3K27ac (histone H3 lysine 27 acetylation) levels on the enhancer and the promoter of its target gene, thereby activating gene expression through interacting with various transcription factors. In this way, the NAT KHPS1 results in local modifications of chromatin structure, which stimulates the binding of transcription factor E2F1 and promoting transcription [51].

In addition to mediating histone modifications, NATs have been shown to shape DNA methylation patterns. As a tumor suppressor gene, PTEN is mutated and epigenetically silenced in various cancer types [52–55]. The antisense transcript of PTEN can recruit DNA methyltransferase 3a (DNMT3a) to the PTEN promoter, leading to chromatin condensation and ultimately epigenetic and transcriptional suppression of PTEN expression [56] (Fig. 2b).

### Transcriptional interference

Antisense transcription can modulate the expression of sense transcript through transcriptional interference [12], in which one transcriptional process exerts a suppressive effect on another in cis and directly [57, 58].

**Fig. 3:**
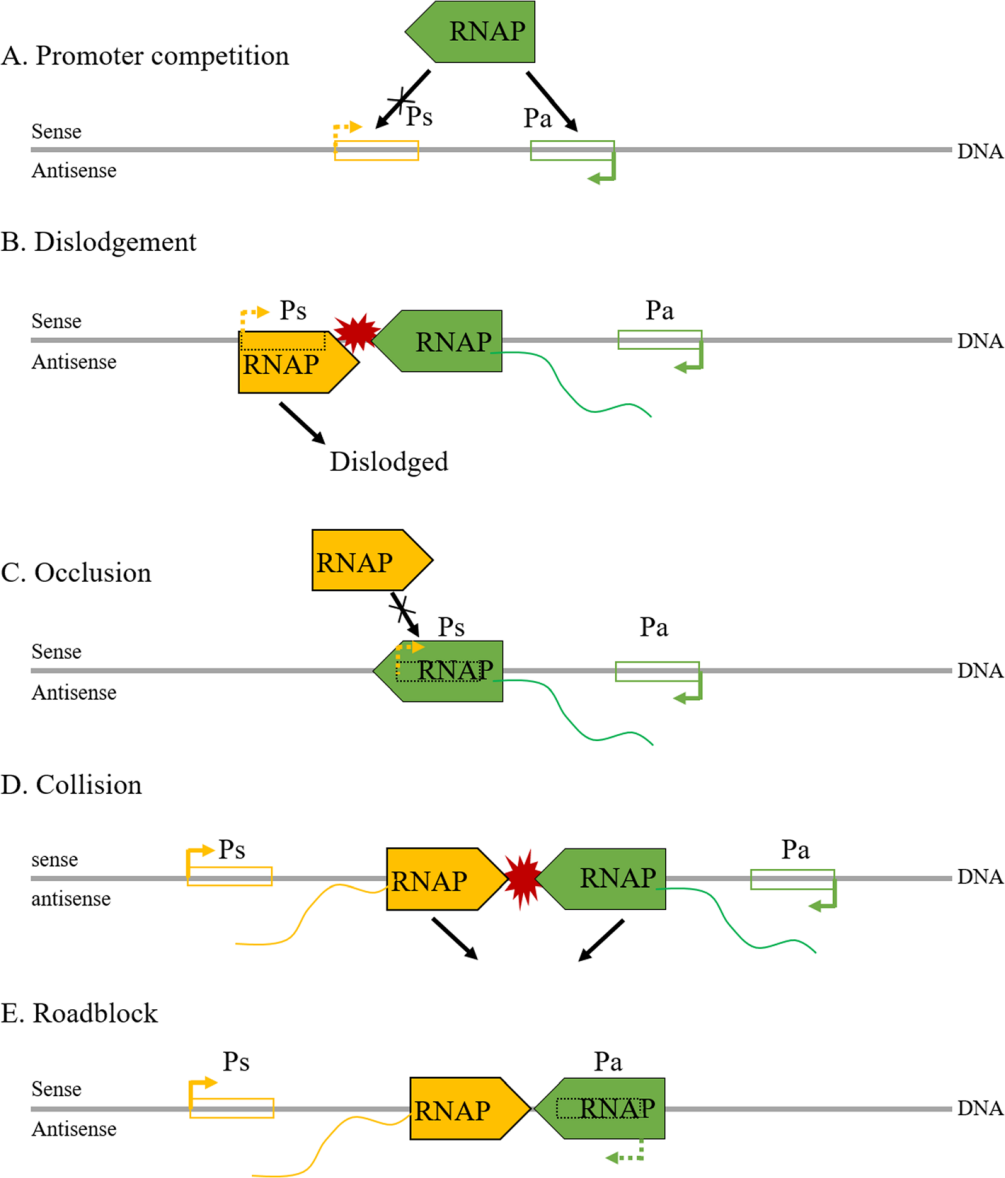
NATs in transcriptional interference. In the initiation phase: **a** Promoter competition, the promoters of sense and antisense genes compete for RNAP complex; **b** Dislodgement, the RNAP complex of the sense gene promoter is dislodged by the arrival of the antisense gene RNAP complex; **c** Occlusion, the promoter of the sense gene is occluded by the antisense RNAP complex during antisense transcript elongation. In the elongation phase: **d** Collision, RNAP complexes collide in the overlapping region of the sense and antisense genes, blocking further transcriptions; **e** Roadblock, the antisense gene RNAP complex blocks that of the sense gene promoter. Ps, promoter of sense gene; Pa: promoter of antisense gene

Antisense-mediated transcriptional interference may take place not only in transcription initiation but also in transcription elongation, and several theoretical models have been established by Shearwin and colleagues to enlighten the underlying mechanisms: (A) Promoter competition, occupation of RNA polymerase (RNAP) by the antisense promoter inhibits its occupation by the sense promoter, thus downregulating both transcripts; (B) Dislodgement, the sense promoter is sensitive to RNAP binding, but slow to initiate, and thus be dislodged by the antisense RNAP complex; (C) Occlusion, the sense promoter is occluded by the RNAP complex during elongation of the antisense transcript; (D) Collision, a collision between RNAP complexes in the overlapping region of the sense and antisense genes blocks further transcriptions, and (E) Roadblock, RNAP bound to the antisense promoter blocks the progress of the RNAP complex from the sense promoter. The first 3 of these models may participate in the transcription initiation phase of sense genes, and the final 2 in the elongation phase (Fig. 3). For more details, please refer to [58]. However, it is unlikely that transcriptional interference can be fully explained by such simple models. The sophistication of chromatin structure and the diversity of transcription initiation complexes pose challenges for studying transcriptional interference in higher eukaryotes.

Nevertheless, accumulating evidence has demonstrated that NATs can regulate transcriptional interference. AIRN is the antisense noncoding RNA of IGF2R, an imprinted tumor suppressor [59, 60]. AIRN can suppress the expression of the IGF2R promoter. Further studies have revealed that the transcriptional overlap between AIRN and the IGF2R promoter silences IGF2R, rather than the AIRN products, indicating the involvement of AIRN in transcriptional interference [59]. Another example is GNG12-AS, which is transcribed in an antisense orientation to the tumor-suppressor DIRAS3. In this case, GNG12-AS, preferentially localized in the nucleus, can be transcriptionally silenced via a specific siRNA (which can exert regulatory effects on transcription in the nucleus in the presence of AGO2 [61, 62]) complementary to GNG12-AS1 exon 1, which is adjacent to the TSS. Mechanistically, the specific siRNA can form a regulatory complex with AGO2 and GNG12-AS1 at the GNG12-AS1 TSS to inhibit RNAP recruitment, leading to the repression of transcription initiation and elongation of GNG12-AS1. In contrast, a concomitant increase in RNAP recruitment is observed at the DIRAS3 TSS, followed by the overexpression of DIRAS3 [63], suggesting the promoter competition model of transcriptional interference.

### NATs mediate alternative splicing

Alternative splicing is a process by which a single gene can produce multiple protein isoforms that may exert different or even opposite functions on biological behaviors [64]. Classically, NATs participate in the regulation of alternative splicing by interacting with specific splicing factors [65]. EGOT is an antisense ncRNA transcribed from sense ITPR1, which induces autophagy and sensitizes breast cancer cells to paclitaxel (an anticancer agent that prevents mitosis and inhibits cancer cell proliferation [66]). EGOT can recruit a splicing factor, hnRNPH1, through specific motifs and bind to the ITPR1 pre-mRNA to regulate the alternative splicing of pre-ITPR1, thereby leading to ITPR1 overexpression. In this manner, EGOT acts as an oncogene [67].

In addition, NAT can directly disrupt the balance among splicing variants by forming an RNA-RNA duplex with its sense RNA to mask splicing sites [11]. A well-known example is ZEB2-AS1, the NAT of ZEB2. ZEB2 is a transcription factor that participates in cancer complexity and functions as a biomarker for endothelial-mesenchymal transition (EMT). ZEB2-AS1 overlaps with the 5’ UTR (untranslated region) intron of ZEB2, which contains an internal ribosome entry site (IRES) necessary for ZEB2 translation, thereby forming an RNA-RNA duplex to protect the IRES from processing, and consequently enhancing the translation efficiency of ZEB2 [68]. This is thought to drive EMT in various cancers [69].

NATs can also affect alternative splicing by fine-tuning chromatin signatures. A nuclear antisense lncRNA, transcribed from the FGFR2 locus, recruits polycomb-related proteins and KDM2a, a histone demethylase, to the FGFR2 locus, leading to chromatin remodeling, thereby preventing the binding of a chromatin-splicing adaptor complex necessary for FGFR2 splicing [70].

### NATs mediate sense RNA editing

RNA editing is a widespread post-transcriptional mechanism by which specific nucleotides in an RNA sequence are modified. Adenosine deaminases that act on RNA (ADAR) proteins are key regulatory enzymes responsible for adenosine-to-inosine (A-to-I) RNA editing in double-stranded RNA (dsRNA) molecules, which is the most common pattern of RNA editing in humans [71]. Such modifications may be dysregulated in cancer, and alter the expression of the edited transcript [72, 73]. Notably, nuclear NATs commonly form dsRNA structures with their sense counterparts. Identifying the occurrence frequency of RNA editing by NATs might be ambitious, but it would be a worthwhile exploration. Indeed, some NATs have been implicated in A-to-I RNA editing.

PCA3 is an antisense lncRNA transcribed from intron 6 of the PRUNE2 gene, which acts as a tumor suppressor in prostate cancer. PCA3 can bind PRUNE2 pre-mRNA to form a PRUNE2/PCA3 dsRNA structure, which undergoes adenosine ADAR-dependent A-to-I RNA editing, leading to decreased expression of PRUNE2 and increased tumor cell proliferation [71]. Another interesting example of RNA editing by NATs is HIF1A-AS2. Full-length HIF1A-AS2 itself can form multiple RNA editing-favoring dsRNA structures, therefore it is a promising substrate of RNA editing. Further examinations have validated the fact that HIF1A-AS2 undergoes A-to-I editing mediated by ADAR1, an essential member of the ADAR family [74].

### NATs mediate mRNA stability, translatability, and post-translational modifications

As regulators that can be found in almost every biological process, NATs also exert effects on mRNA stability, translatability, and post-translational modifications (PTMs) [75].

In general, human NATs can form RNA duplexes with their sense partners to regulate sense mRNA stability by directly masking specific sequences that could otherwise facilitate mRNA degradation [76]. Specifically, antisense/sense RNA duplex can regulate mRNA degradation by interacting with an RNA interference (RNAi) mediator, an RNA decay-promoting factor, or the RNase digestion machinery. As a critical component of the RNAi machinery, DICER induces gene silencing either via RNA cleavage or translational suppression [77]. ADAR1, a regulator of dsRNA molecules, distinguishes its function in RNAi from RNA editing by forming an ADAR1-DICER heterodimer complex [78]. GLS is an imprinted oncogene that can stabilize c-MYC, another well-defined oncogene [79]. In addition, the intronic antisense transcript of GLS (GLS-AS) can form an RNA duplex with the sense GLS pre-mRNA, which further inhibits the expression of GLS through ADAR1/DICER-dependent RNA degradation and leads to c-MYC downregulation [80]. An example of an RNA decay-promoting factor is HuR, which acts as a powerful regulator of mRNA stability by binding to specific motifs at the UTRs of target mRNAs [81], including PDCD4. PDCD4 and its NAT partner, PDCD4-AS1, suppress breast cancer progression collaboratively. Mechanistically, PDCD4-AS1 prevents the interaction between PDCD4 mRNA and HuR in the nucleus by forming an RNA duplex with PDCD4, stabilizing PDCD4 mRNA and inhibiting malignant phenotypes [82]. Lastly, single-stranded RNA is susceptible to RNase digestion, while the antisense-sense RNA duplex may have a physical protective effect against RNase degradation, consequently gaining mutual stability [75]. In gastric cancer, the lncRNA KRT7-AS1 forms an embedded RNA duplex with its oncogenic sense gene, KRT7, at the exon adjacent to the poly(A) tail, increasing KRT7 mRNA stability and protein expression by protecting it from RNase degradation, consequently promoting tumor cell proliferation and migration [83]. Despite non-coding/protein-coding pairing, a similar mechanism can also be observed in protein-coding/protein-coding pairing. DHPS, a protein-coding NAT of WDR83 mRNA, forms a protective tail-to-tail RNA duplex with WDR83 at the 3′ UTR to enhance mutual stability [15].

Besides, the antisense/sense RNA duplex can directly regulate protein translation through translational interference. For example, BCMA mRNA overlaps with its antisense transcript at the coding sequences. Overexpression of the BCMA NAT significantly decreases BCMA protein levels without changing BCMA RNA levels, indicating that BCMA NAT regulates BCMA expression mainly at translational level [84, 85]. Another example of translational interference by NAT is the antisense transcript of PU.1, which clarifies the underlying mechanism. PU.1 (also known as Spi-1 proto-oncogene) overlaps with its antisense partner at the coding region. PU.1 NAT negatively modulates the expression of PU.1 protein without downregulating of PU.1 mRNA levels. Further efforts have shown that the overlapping coding region may interfere with the interaction between PU.1 mRNA and eEF1A (which promotes protein biosynthesis) [86, 87].

Based on the above findings, we note that the duplexes implicated in mRNA stability are adjacent to or just at the non-coding sequences of sense mRNAs, while the duplexes, which regulate translational interference, are located at the coding regions. Therefore, we propose the preliminary hypothesis that RNA-RNA duplexes may alter the secondary or tertiary structure of mRNA, thereby affecting mRNA stability and mRNA/ribosome interaction, and the location of the overlapping regions may be one of the keys to distinguishing the effects on mRNA stability from translatability. Although the precise mechanisms require further study, we believe that this hypothesis can, at least in part, unravel the mystery.

Another underlying mechanism of NAT-mediated mRNA stability and translatability is miRNA sponging. miRNAs often induce destabilization and translational repression of targeted mRNAs by binding to the 3′ UTR [88]. By acting as ceRNAs to sponge miRNAs, NATs mask the binding sites between miRNAs and target mRNAs to inhibit miRNA-induced functions. For example, ZNFX1 antisense RNA 1 (ZFAS1) may upregulate the expression of BMI1, a major component of epigenetic repressor PRC1, by competitively binding miR-200b/c, thereby driving tumorigenesis in osteosarcoma [89]. NATs can also regulate their cognate sense counterparts and contribute to malignant behaviors through this mechanism. The antisense lncRNA SOX9-AS1 promotes the expression of its sense partner SOX9 at both RNA and protein levels by sponging miR-5590-3p, thereby maintaining and facilitating the oncogenic functions of SOX9 in hepatocellular carcinoma [90].

Additionally, a special type of NATs, termed SINEUPs (SINE element-containing translation UP-regulators), has attracted attention due to their role in promoting the translation efficiency of the cognate sense mRNAs [91]. SINEUP activity was first demonstrated in mice: antisense lncRNA of Uchl1 promoted the translation efficiency of Uchl1 through antisense/sense binding, in which the overlapping region on the antisense transcript contains a SINEB2 domain to drive SINEUPs activity [92]. Similarly, in human cells, NAT of PPP1R12A exerts SINEUP activity to facilitate PPP1R12A translation [93].

Finally, dysregulated PTMs, which mainly consist of phosphorylation and ubiquitination, are profoundly implicated in tumorigenesis and chemoresistance by regulating protein activity and stability [94]. As expected, NATs, which can be found in almost every biological process, also modulate PTMs. ASB16-AS1, a NAT that promotes tumor growth in vivo and attenuates cisplatin-induced cell apoptosis in gastric cancer, has been identified as a regulator of PTM. Specifically, TRIM37 phosphorylation exerts oncogenic functions in gastric cancer. ASB16-AS1 can recruit ATM, a protein kinase, to TRIM37 by forming physical bindings with ATM and TRIM37, inducing TRIM37 phosphorylation and synergistically facilitating malignant phenotypes [95]. An example of ubiquitination is HOTAIR. HOTAIR can bind to RUNX3 protein, which functions as a tumor suppressor in multiple malignancies, and promote the interaction between RUNX3 and the E3 ubiquitin ligase MEX3B, inducing degradation via MEX3B-mediated ubiquitination [31, 96].

### NATs in the hallmarks of cancer

**Fig. 4:**
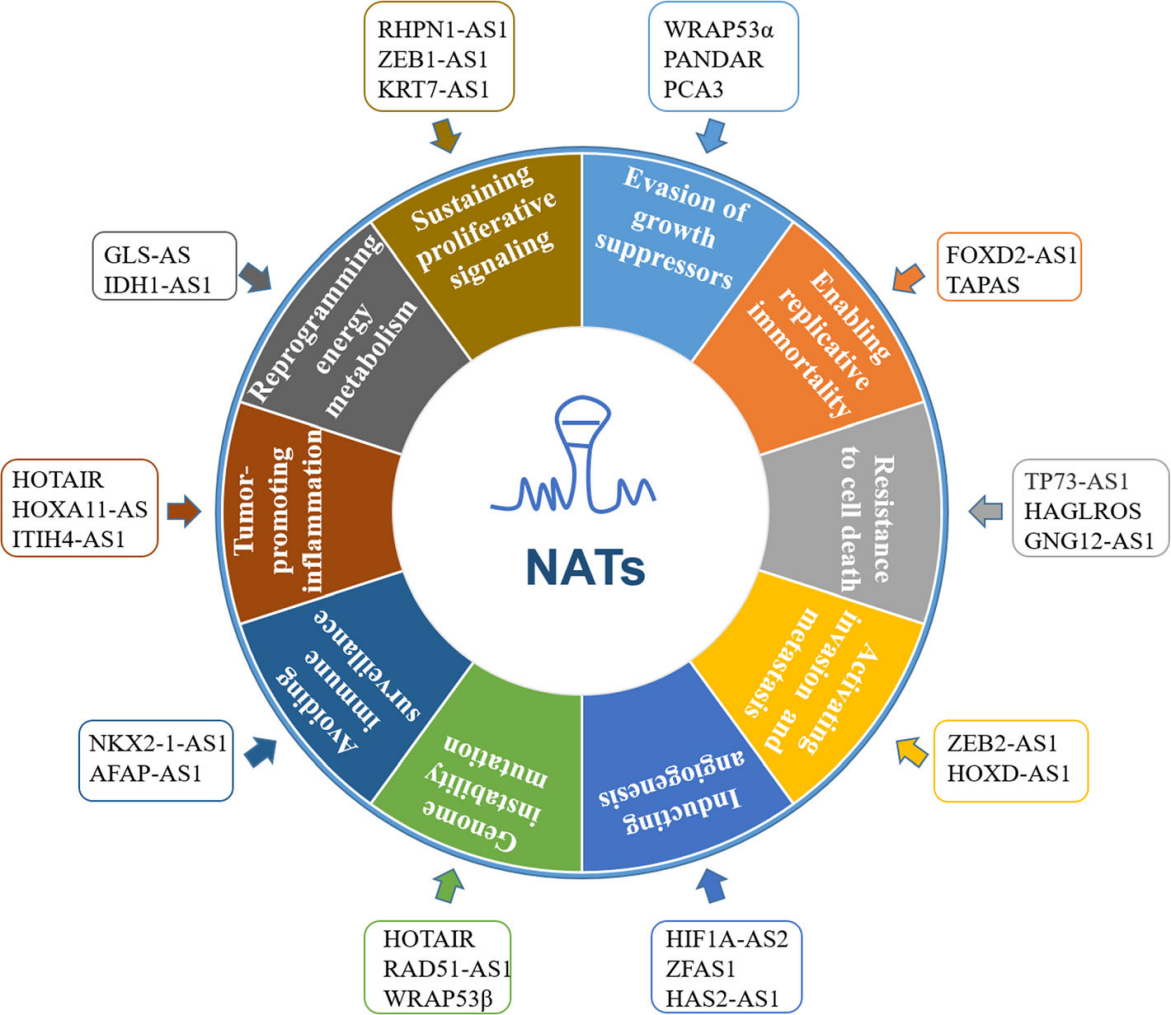
NATs contribute to each of the hallmarks of cancer

Carcinogenesis is a multistage process that results from dysregulated intracellular networks that unlocks a Pandora’s box of disease and from intercellular communication that hides malignancy by orchestrating the tumor microenvironment (TME). In this manner, the integrated intracellular and intercellular cross-talk contributes to the hallmarks of cancer, as described by Hanahan and Weinberg in 2011 [22], and ultimately drives malignant phenotypes [97, 98]. NATs extensively participate in the hallmarks of cancer (Fig. 4) via the abovementioned molecular mechanisms, either separately or simultaneously. Here, we discuss the emerging roles of NATs in the hallmarks of cancer to demonstrate an up-to-date understanding of this rapidly developing field. While we did our best to cover this topic, some additional publications may also help get the full picture [99–102].

### Sustaining proliferative signaling

Normal cells are regulated by growth signals and inhibitory factors to maintain homeostasis and the architecture and functions of tissues and organs. In contrast, cancer cells employ a variety of mechanisms to reduce their dependence on external growth signals, leading to sustained proliferation: (1) Producing growth factors and cognate receptors themselves to stimulate autocrine proliferation. (2) Stimulating tumor-associated stroma, which in turn produces growth factors, achieving sustained proliferation in a paracrine manner. (3) Promoting the expression of growth factor receptors, rendering cancer cells hyper-responsive to proliferative signals. (4) Altering or destroying proliferative signaling pathways, leading to cancer cells escaping the restraint of exogenous proliferative signals [22, 102]. As ubiquitous regulators, NATs have demonstrated their important roles in sustaining cancer cell proliferation. RHPN1 antisense RNA1 (RHPN1-AS1) upregulates the expression of fibroblast growth factor 2 (FGF2), a potent regulator of mitogenic activity [103], by binding to miR-299–3p, thereby promoting cervical cancer cell proliferation [104]. Apart from regulating growth factor expression, NATs can also promote proliferation by modulating receptor abundance and the downstream signaling pathways. EGFR (epidermal growth factor receptor) and HER2, members of the EGFR family, play critical roles in sustaining cancer cell proliferation, aided by the downstream ERK1/2 signaling pathway. In hepatocellular carcinoma, the NAT MYLK-AS1 can increase EGFR and HER2 expression and subsequently trigger the ERK1/2 signaling pathway, leading to increased cancer cell proliferation [105].

### Evasion of growth suppressors

Tumor suppressor genes, known as the guardians of the genome, are in charge of the fate of the cell by regulating the cell cycle and apoptosis [106]. Therefore, cancer cells must escape these genes to survive and progress, in addition to the induction and maintenance of growth-stimulating signals. TP53 and PTEN, two canonical tumor suppressors, are key nodes in the biological networks implicated in the evasion of growth suppressors [107, 108]. Accumulating evidence shows that NATs-mediated TP53 and PTEN dysregulation participate in the evasion of cancer cells from tumor-suppressing functions. PANDAR (promoter of CDKN1A antisense DNA damage activated RNA) can interact with its binding protein SFRS2, a splicing factor that negatively regulates TP53 expression and phosphorylation, thereby facilitating the evasion of growth suppressor in ovarian cancer [109]. Other than PANDAR, the NAT WRAP53α also regulates TP53. Specifically, WRAP53α overlaps with the 5′ UTR of TP53 mRNA to form an antisense/sense duplex, thereby protecting TP53 from degradation and consequently increasing TP53 mRNA and protein levels. Through this mechanism, silencing WRAP53α attenuates TP53-induced apoptosis in breast cancer cells carrying wild-type TP53 [110]. PTEN can be epigenetically silenced by DNMT3a-mediated methylation under the guidance of its antisense counterpart [56].

### Enabling replicative immortality

Apart from interfering with growth signals, cancer cells also harbor the characteristic of replicative immortality [102]. The telomere, located at the end of chromosome, is critical for the replication limits in normal cells. The telomere shortens gradually as the number of cell divisions increases. Hence, the telomere length indicates and further dictates the potential of cell replication. In contrast, cancer cells can circumvent the replication barrier posed by telomere shortening mainly by overexpressing telomerase, an enzyme that can maintain telomere length, and thereby gain the characteristic of replicative immortality [111, 112]. Recent studies have indicated that specific NATs may be involved in regulating telomerase expression. As an imprinted oncogenic NAT, FOXD2-AS1 can upregulate the expression of telomerase reverse transcriptase (TERT), the catalytic subunit of telomerase, by sponging miR-7-5p, maintaining and facilitating cancer stem cell phenotypes in thyroid cancer [113]. Another interesting finding is that TERT itself has an antisense partner, termed TERT antisense promoter-associated (TAPAS) RNA. In human embryonic stem cells, immortalized B-cells, and several primary tumor samples, TAPAS expression correlates negatively with TERT expression, implying a role for TAPAS in modulating replicative immortality [114].

### Resistance to cell death

Unlimited replication does not ultimately lead to immortality. Therefore, another hallmark of cancer cells is the ability to successfully circumvent the cell death barrier, in addition to maintaining a continuous state of division. As controlled cell death processes, apoptosis and autophagy are considered essential for maintaining the homeostasis of cell death [115, 116]. The PI3K/AKT/mTOR pathway has long been identified as a powerful intersection between the regulatory networks of apoptosis and autophagy [22, 117]. The PI3K/AKT/mTOR pathway is often deregulated in cancer [118]; notably, accumulating evidence demonstrates that specific NATs can regulate the pathway to shape the malignant phenotypes that facilitate the resistance to cell death. In drug-resistant breast cancer cells, HOTAIR can downregulate the resistance by attenuating the PI3K/AKT/mTOR pathway [119]. In clear cell renal cell carcinoma, TP73-AS1 inhibits the transcription of KISS1, a negative regulator of the PI3K/AKT/mTOR pathway, by recruiting EZH2, the catalytic subunit of PRC2, thereby reducing cell death [120]. In colorectal cancer cells, HAGLROS (HOXD antisense growth-associated lncRNA) regulates apoptosis and autophagy to resist cell death; the PI3K/AKT/mTOR pathway and ATG5, a biomarker of autophagy, are the possible functional targets of HAGLROS [121]. Apart from the PI3K/AKT/mTOR pathway, NATs can also mediate other molecules directly or indirectly associated with resistance to cell death. Compelling functional studies have validated this in numerous cancer types, such as the involvement of the NATs ZEB1-AS1 [42], PANDAR [109], and WRAP53α [110].

### Activation of invasion and metastasis

When cancers progress from residency in epithelial tissues to higher pathological grades of malignancy, they exhibit characteristics of local invasion and distant metastasis. Frequently, cancer cells enter the invasion-metastasis cascade first by invading the barrier of cell-cell/cell-matrix adhesion, in which EMT is prominently implicated [122, 123]. As a pivotal biomarker of cell-cell adhesion, E-cadherin maintains the integrity of epithelial cell layers and often exerts a tumor-suppressive function. The loss of E-cadherin drives cancer cell invasion and metastasis by modulating EMT, while the upregulation of E-cadherin attenuates such phenotypes [124]. There are various means of regulating E-cadherin, which include ZEB2-AS1 and HOTAIR. Specifically, ZEB2-AS1 overlaps with the intron of the ZEB2 5′ UTR, in which contains an IRES, enhancing the translation efficiency of ZEB2, a transcriptional suppressor of E-cadherin, and consequently leading to the downregulation of E-cadherin in breast cancer [68]. In oral squamous cell carcinoma, HOTAIR recruits EZH2 to induce H3K27me3 of the E-cadherin promoter and thus silences E-cadherin expression, consequently potentiating invasion and metastasis [125]. Moreover, HOTAIR is often overexpressed and may serve as a promising biomarker of distant metastasis in various cancer types [24, 125, 126].

EMT is activated not only by the disruption of cell-cell adhesion, but also by the cell-matrix crosstalk, in which the matrix metalloproteinases (MMPs) play an important role in inducing breakdown of the extracellular matrix and subsequently paving the way for cancer cell invasion and metastasis [123, 127]. MMP9, an MMP family member [127], is overexpressed in multiple cancers and can be regulated by various NATs. In non-small cell lung cancer, HOXD-AS1 promotes MMP9 expression by sponging miR-133b, facilitating migration and invasion [128]. In ovarian cancer, TP73-AS1 potentiates metastasis by upregulating MMP9 [129]. These are simple examples, but they also provide solid evidence for NATs involvement in EMT. Future studies on identification of such NATs may broaden the scope of regulation of invasion and metastasis.

### Induction of angiogenesis

Blood vessels play an important role in maintaining tissue and cell functions, allowing the import of nutrients and oxygen, and the export of metabolic waste and carbon dioxide [130]. In general, angiogenesis is restricted to embryogenesis. In adults, under certain conditions, such as female reproductive cycling and wound healing, the so-called angiogenesis switch can be switched on transiently. In contrast, angiogenesis is switched on and remains on in cancer, which leads to the constant growth of new vessels for sustaining tumor growth [22, 123]. Also, as the Chinese proverb states, “Before soldiers and horses move, provisions and grass go first,” cancer cells therefore must control the angiogenesis switch to provide a precondition for tumorigenesis and progression [131]. As the most intensively studied proangiogenic pathway, the HIF1A/VEGFA (hypoxia-inducible factor 1-alpha/vascular endothelial growth factor A) pathway is strengthened in various malignancies, and its strength correlates with multiple malignant phenotypes [132, 133]. NATs also regulate the angiogenesis hallmark by interacting with the HIF1A/VEGFA pathway. HIF1A-AS2, a NAT of HIF1A, also known as aHIF, was previously considered a negative regulator of HIF1A in a computer analysis model. In this case, the HIF1A 3′ UTR may present as a hairpin structure, containing AU-rich elements (AREs) that may induce mRNA decay. HIF1A-AS2 may also overlap with the HIF1A 3′ UTR, thereby unfolding the hairpin structure to facilitate HIF1A mRNA degradation [134]. A few studies have discussed this hypothesis, such as in non-small cell lung cancer [135] and ovarian cancer [136], but it has not been validated experimentally. Despite that, HIF1A-AS2 can promote HIF1A expression and regulate the HIF1A/VEGFA pathway by sponging miR-548c-3p, exerting a proangiogenic function in breast cancer [137]. In bladder cancer samples following treatment with cisplatin (a chemotherapy drug), HIF1A-AS2 and HIF1A are both overexpressed and correlate positively [138]. Therefore, HIF1A-AS2 might regulate HIF1A expression in a tissue-specific manner. Further studies are needed to reveal the underlying mechanisms. Additionally, other NATs can also regulate angiogenesis by interacting with the HIF1A/VEGFA pathway, either as an upstream regulator (e.g., ZFAS1 [139]) or as a downstream effector (e.g., HAS2-AS1 [140]).

### Genome instability and mutation

Cancer is a genetic disease; genomic alteration is considered the trigger of the hallmarks described above [22]. Briefly, genomic alteration may confer selective advantages on cellular growth, and cell viability and motility, enabling tumorigenesis and progression. Genome instability and mutation are the main forms of genomic alteration. As discussed earlier, epigenetic modifications induce genome instability via DNA methylation and histone modifications, leading to epigenetic repression of target genes and consequently being implicated in malignant phenotypes. As NATs are predominantly located in the nucleus, it is not surprising to find that they play a role in epigenetic mechanisms. Still taking HOTAIR as an example, despite it being able to induce H3K27me3 to silence target genes, HOTAIR can also suppress H3K27ac to achieve epigenetic repression. In gastric cancer, HOTAIR-mediated dynamic homeostasis between H3K27me3 and H3K27ac regulates the stability and transcription of the E-cadherin gene. Specifically, knockdown of HOTAIR attenuates H3K27me3 of the E-cadherin promoter and derepresses H3K27ac, thereby increasing E-cadherin expression and consequently inhibiting cancer cell EMT [125]. In addition, telomere shortening also facilitates genome instability. In this case, additional telomere shortening may induce telomere crisis, which compromises the protective effect of the telomere on the chromosome end, ultimately causing extensive genome instability to drive tumorigenesis. Telomerase, which restores telomere length, is therefore critical for avoiding telomere crisis and maintaining genome integrity [141–143]. However, as described above, telomerase overexpression enables replicative immortality, indicating the double-edged effects of telomerase on the hallmarks of cancer. Therefore, NATs such as FOXD2-AS1 and TAPAS, which regulate telomerase expression, may also regulate genome stability. Further studies on identifying NATs that regulate telomerase and on balancing the advantages and disadvantages of telomerase may shed light on NAT-mediated genome alteration.

Furthermore, genome instability contributes to mutations of the hallmark-enabling genes under the condition of genome maintenance machinery dysfunction. RAD51 is a hub protein involved in the DNA repair machinery through homologous recombination. Malfunction of RAD51 is considered as a critical event driving genome instability and tumorigenesis [144]. In hepatocellular carcinoma cells, RAD51 has a negative correlation with its antisense partner, RAD51-AS1. Silencing RAD51-AS1 increases RAD51 protein expression significantly without altering RAD51 mRNA levels [145]. Moreover, this is not the only example of NAT-mediated genome mutations. In glioblastoma, HMMR-AS1 interferes with the translation of RAD51 and ATM (a master controller of DNA repair) [146]. WRAP53β (also known as WDR79), another TP53 NAT independent of WRAP53α, mediates DNA repair with the participation of RAD51 and ATM in various cancer cell lines [147]; in addition, the expression of WRAP53β protein correlates positively with TP53 mutations in non-small cell lung cancer samples [148]. Although the underlying mechanisms are not known, these findings provide concrete evidence that NATs can be involved in the machinery of genome mutations.

Notably, NATs themselves may give rise to functional mutations. The lncRNA SIRT1-AS can increase the stability of its sense counterpart, SIRT1 mRNA (a histone deacetylase that deeply participates in genome stability maintenance [149], and EMT [150]), by sponging miR-29c, facilitating hepatocellular carcinoma cell proliferation. However, a single-nucleotide mutation has been observed in SIRT1-AS; it decreases the risk of hepatocellular carcinoma possibly by altering the secondary structure of SIRT1-AS and preventing binding to SIRT1 mRNA [151]. Taken together, these NATs depict a preliminary outline of their promising potential in regulating genome instability and mutation.

### Avoidance of immune detection and destruction

Under constant surveillance of the immune system, human body maintains a dynamic homeostasis to provide protection against tumor. Therefore, disrupting the equilibrium and avoiding identification and/or elimination by the immune surveillance is a primary task in tumor survival [22, 152]. Notably, genome instability and mutations result in tumor heterogeneity, endowing different cancer cell subclones with varying sensitivity to immune surveillance [152]. The immune system can detect, target, and destroy sensitive cancer cells, while non-sensitive cancer cells are invisible to immune surveillance, thereby achieving the tasks of surviving and thriving. From this perspective, the immune system is a double-edged sword, not only exerting an antitumor effect, but also inadvertently selecting tumor variants that are undetectable and indestructible by the immune system, consequently promoting tumor evolution. Further, the immune checkpoints render cancer cells invisible. Programmed cell death 1 (PD1) and PD1 ligand 1 (PD-L1), the well-defined immune checkpoints that exert stimulatory or inhibitory functions on the immune response, are considered the key to the disequilibrium [153]. NAT-mediated dysregulation of PD1/PD-L1 induces immune tolerance, enabling cancer cells to escape from immune detection and destruction. In lung cancer cells, NKX2-1-AS1, the NAT of the transcription factor NKX2-1, is a negative regulator of PD-L1 mRNA and protein levels. Further examinations have validated the fact that NKX2-1-AS1 acts as a decoy to prevent NKX2-1 from binding the PD-L1 promoter, thereby decreasing PD-L1 transcription, and ultimately leading to attenuated evasion of the immune system [154]. Another NAT, AFAP-AS1, correlates positively with PD1 in nasopharyngeal carcinoma [155]. These examples indicate that NATs may play a role in regulating immune checkpoints. Further efforts on: (1) identifying of NATs that participate in the immune surveillance networks; (2) exploring the functions and underlying mechanisms of immune-NATs; (3) applying immune-NAT targeted molecules in cancer therapy, either alone or in combination with immunotherapy, will greatly deepen our understanding of the sophisticated interaction between NATs and immune surveillance. In addition, escape from immune surveillance does not occur independently; it is closely intertwined with tumor-associated inflammation, which is discussed in the next section.

### Tumor-promoting inflammation

Inflammation goes through every stage of tumorigenesis and progression [156]. Together with cancer cells, stromal cells, and immune cells, inflammatory cells can shape malignant characteristics and phenotypes by orchestrating an inflammatory TME [156, 157]. Functionally, inflammation acts as a double-edged sword in cancer, much like the immune system. Simply put, inflammation can induce, and vice versa, genome instability and oncogenic mutations and compromise the immune surveillance system, creating a tumor-promoting TME to enrich malignant transformation and accelerate tumor progression by directly releasing oncogenic signals and/or interacting with other TME components. On the other hand, inflammation can reinforce immune surveillance by enhancing immune cell infiltration into tumors, thereby switching TME to a tumor-suppressing direction [158–160]. The dynamic crosstalk between the tumor-promoting and tumor-suppressing signals is, to some extent, a master controller of tumorigenesis. It is well established that nuclear factor-κB/signal transducer and activator of transcription 3 (NF-κB/STAT3), the well-defined regulatory hubs of tumor-associated inflammation, play pivotal roles in maintaining the delicate crosstalk. Dysregulation of NF-κB/STAT3 will upset the delicate balance, leading to a predisposition to the pro-tumorigenic direction and ultimately driving tumor initiation and progression [161–163]. Therefore, it is important to fully uncover the sophisticated networks of NF-κB /STAT3 and to determine how they can be fine-tuned to exert anti-tumorigenic functions. NATs, which have demonstrated their roles in tumor-associated inflammation, may provide insights into the networks. HOTAIR is overexpressed in ovarian cancer, which induces NF-κB activation and the subsequent secretion of IL-6 (an NF-κB downstream molecule that activates STAT3, also playing a critical role in reprogramming the TME [164]) by suppressing Iκ-Bα (an NF-κB inhibitor). Furthermore, NF-κB exerts its functions as a transcription factor to bind the promoter of HOTAIR, thus forming an NF-κB-HOTAIR positive feedback loop and driving malignant phenotypes [165]. Additional studies have shown that HOTAIR can directly or indirectly regulate STAT3 in a reciprocal manner in multiple malignancies [30, 166, 167]. HOTAIR is not the only example of NAT-mediated tumor-associated inflammation. Briefly, HOXA11-AS regulates STAT3 expression by acting as a miRNA sponge in liver cancer, thereby accelerating tumor growth and metastasis [168]. In colorectal cancer, ITIH4-AS1 facilitates tumorigenesis and progression by modulating STAT3 phosphorylation [169]. These investigations present credible clues for NAT regulation of NF-κB/STAT3, and shed light on tumor-associated inflammation.

### Reprogramming energy metabolism

Metabolism fuels life, whether in cancer cells or in normal cells. However, metabolism in cancer cells has distinct characteristics when compared with that in normal cells. Although the underlying mechanisms of this phenomenon remain to be unveiled, the Warburg effect may provide inspiring insights. The Warburg effect refers to the phenomenon where, regardless of sufficient oxygen, cancer cells reprogram their metabolism phenotypes to acquire a preference for glycolysis [170, 171]. Through glycolysis, cancer cells produce the glycolytic intermediates necessary for rapid proliferation [172] and create an acidic microenvironment favorable for EMT [173]. Accordingly, the Warburg effect caused by the reprogrammed metabolism can shield the onset and progression of cancer. From this perspective, cancer can also be considered a metabolic disease. Mechanistically, cancer cells flip the metabolic switch largely by modulating central metabolic molecules, such as c-MYC [174] and HIF1A [175]. Notably, c-MYC cooperates with HIF1A to promote glycolysis in response to hypoxia [174]. As discussed earlier, c-MYC can be regulated by GLS-AS in an RNAi-dependent manner, and the correlation of HIF1A to multiple NATs has been demonstrated. Therefore, it would be intriguing to explore whether NATs are involved in the cooperation between c-MYC and HIF1A. IDH1-AS1 is a NAT transcribed from the opposite direction of IDH1, which encodes a key enzyme in metabolism and induces HIF1A degradation by forming a homodimer structure. IDH1-AS1 can physically bind IDH1 to promote its homodimerization, leading to increased IDH1 enzymatic activity and consequently accelerating HIF1A degradation. c-MYC can decrease IDH1 activity through transcriptional inhibition of IDH1-AS1. In this manner, IDH1-AS1 acts as a bridge between c-MYC and HIF1A and attenuates the activation of the Warburg effect [176].

Notably, as the enabling characteristics, destabilized genome, disequilibrated immune surveillance, tumor-associated inflammation, and reprogrammed metabolism are complementary and intricately intertwined [22, 123]. They coordinate with each other to create the cancer seed and fertilize the soil. Protein is the executor of biological function [177], while NATs mainly consist of ncRNAs [12]. In addition, NATs have not attracted much attention. Altogether, this renders NATs obscure. Despite these concerns, NATs have proven to be powerful, yet sidelined, regulators, fine-tuning every hallmark of cancer.

### Therapeutic promise in targeting NATs

NATs are ubiquitous in all branches of life and deeply participate in the diverse hallmarks of cancer. The expression of NATs generally demonstrates a highly cell- and/or tissue-specific pattern [178], making NATs promising candidates for therapeutic targets. Multiple approaches have been explored for targeting NATs: (1) RNAi by using siRNAs, shRNAs, and miRNAs. RNAi reduces RNA stability or mRNA translatability in an AGO2-dependent cleavage manner [77]. Contrary to AGO2, which is predominantly expressed in the cytoplasm, NATs are preferentially located in the nucleus [179]. Therefore, the effect of RNAi-mediated knockdown on NATs may be compromised. (2) Antisense oligonucleotides (ASOs). Typically, ASOs are synthetic antisense oligonucleotides that may form a DNA-RNA hybrid with target RNA and thereby induce RNase H-mediated RNA degradation. As RNase H is predominantly expressed in the nucleus, ASOs have a significant advantage over RNAi in targeting nuclear NATs. Particularly, the ASOs designed for inhibiting cis-NATs are termed antagoNATs [178], which may block the interaction between antisense and sense transcripts and subsequently result in NAT degradation and transcriptional derepression of the overlapping/neighboring sense counterparts. Although RNAi- and ASO-based therapies have attracted keen interest in loss-of-function studies on NATs, practical obstacles remain to be solved before clinical application can be achieved, such as off-target effects, insufficient cellular uptake, and cytotoxicity [77, 180]. Hopefully, the development of gene-editing technologies has emerged as an alternative approach for NAT-targeted therapy. (3) Clustered regularly interspaced short palindromic repeats (CRISPR)/Cas9. Cancer is a genetic disease. Correcting pathogenic gene aberrations using gene-editing technologies may be a promising therapeutic strategy in the battle against cancer. As a versatile gene-editing tool, CRISPR/Cas9 makes it possible, under the guidance of single guide RNA (sgRNA), to achieve knock-out/knock-in or transcriptional inhibition/activation of NATs involved in the hallmarks of cancer [181, 182]. Compared with RNAi and ASOs, CRISPR has reduced off-target effects, better efficacy, and consistent activity. However, many NATs overlap with or are adjacent to other genes, and this may hinder the specificity of CRISPR/Cas9-mediated genetic manipulation. A genome-wide study found that only 15% of antisense lncRNAs could be edited safely by CRISPR [183]. We speculate that high-specificity sgRNA is a precondition for CRISPR, and NATs without any intersection with other genes might be more amenable to CRISPR. (4) Other approaches, such as viruses, nanomedicine, morpholinos, and small molecules [180, 184], may also shed light on NATs-targeted therapy through various mechanisms.

Taken together, these approaches have their intrinsic advantages and disadvantages and may complement each other. A reasonable strategy might be choosing the appropriate approach or combination approaches according to the cellular localization of the target NAT.

## Conclusions

NATs are dysregulated in various cancers and are implicated in multiple malignant phenotypes. Therefore, comprehensive understanding of their features and mechanisms of functions is critical for appreciating the importance of this particular class of molecules. Recent studies demonstrate that NATs are emerging as important players in the hallmarks of cancer via their involvement in diverse stages of gene expression regulation, from controlling epigenetic modifications to modulating mRNA PTMs. Indeed, several preclinical experiments have demonstrated promising results after targeting NATs using RNAi-, ASO-, or CRISPR-based approaches [168, 178, 185]. We are just beginning to unravel the mystery of their generation, functions, and mechanisms, and exploring their potential as diagnostic biomarkers and therapeutic targets.

Despite these exciting advancements, the limitations and challenges should not be ignored. First, NATs generally demonstrate relatively low abundance compared with their sense counterparts [186], rendering their annotation challenging. However, the relative low expression and preferential nuclear localization of NATs implies that NATs mainly act in cis as transcriptional regulators, and that small changes may make a big difference. Further investigation is needed to evaluate these possibilities comprehensively. Second, most NATs exhibit limited conservation across species. The investigation of NATs may therefore be obstructed by preclinical evaluation in animal models. Lastly, the overlap between NATs and their sense counterparts may hamper the specificity and safety of NAT-targeted therapy via off-target effects and cytotoxicity. A detailed bioinformatics analysis for eliminating non-specific binding and designing multiple sequences to target different regions of NAT may minimize these adverse effects. However, such approaches are not always practical.

In conclusion, significant evidence of NATs in the cancer hallmarks is being uncovered constantly despite the above limitations and challenges. We have just begun to scratch the surface of NATs. We believe that our knowledge of gene regulation by NATs will continue to expand. Further studies for clarifying their underlying mechanisms of function and for exploring appropriate therapeutic targets will lead to a novel and promising avenue for cancer therapy.

## Abbreviations

NATs: Natural antisense transcripts
ncRNA: Non-coding RNA
lncRNA: Long non-coding RNA
TSS: Transcriptional start site
H3K27me3: Histone H3 lysine 27 trimethylation
H3K27ac: Histone H3 lysine 27 acetylation
HATs: Histone acetyltransferases
ceRNA: Competing endogenous RNA
DNMT3a: DNA methyltransferase 3a
RNAP: RNA polymerase
EMT: Endothelial-mesenchymal transition
UTR: Untranslated region
IRES: Internal ribosome entry site
dsRNA: Double stranded RNA
A-to-I: Adenosine-to-inosine (A-to-I)
PTMs: Post-translational modifications
RNAi: RNA interference
SINEUPs: SINE element-containing translation UP-regulators
TME: Tumor microenvironment
EGFR: Epidermal growth factor receptor
HAGLROS: HOXD antisense growth-associated lncRNA
HIF1A: Hypoxia-inducible factor 1-alpha
VEGFA: Vascular endothelial growth factor A
TERT: Telomerase reverse transcriptase
TAPAS: TERT antisense promoter-associated
MMPs: Matrix metalloproteinases
AREs: AU-rich elements
PD1: Programmed cell death 1
PD-L1: Programmed cell death 1 ligand 1
ASO: Antisense oligonucleotides
CRISPR: Clustered regularly interspaced short palindromic repeats
sgRNA: Single guide RNA

## Availability of data and materials

Data sharing not applicable to this article as no datasets were generated or analyzed during the current study.
